# Integrating single-cell RNA sequencing and Mendelian randomization analysis to identify potential drug targets for dilated cardiomyopathy

**DOI:** 10.1186/s41065-025-00539-9

**Published:** 2025-10-16

**Authors:** Ruikang Liu, Yiying Liu, Chao Meng, Jun Li, Hui Wang, Xuanchun Huang, Shiyi Tao, Xiao Xia, Lilan Su, Yonghao Li, Weiming Fan, Chiyun Sun

**Affiliations:** 1 Guang’anmen Hospital, China Academy of Chinese Medical Sciences, Beijing, China; 2 Graduate School, Beijing University of Chinese Medicine, Beijing, China; 3 Wangjing Hospital, China Academy of Chinese Medical Sciences, Beijing, China; 4 Hunan University of Traditional Chinese Medicine, Changsha, 410208 Hunan China

**Keywords:** Mendelian randomization, Dilated cardiomyopathy, Single-cell RNA sequencing, Druggable genome, Bayesian co-localization

## Abstract

**Background:**

Dilated cardiomyopathy (DCM), a leading cause of heart failure and sudden cardiac death, lacks therapies targeting disease progression. Genome-wide association studies (GWAS) have identified genetic loci linked to DCM, but translating these findings into actionable drug targets remains challenging. Integrating the druggable genome with multi-omics approaches offers a promising strategy for precision therapy.

**Methods:**

We combined Mendelian randomization (MR), Bayesian co-localization, and single-cell RNA sequencing to identify causal drug targets for DCM. Tissue-specific cis-eQTL and pQTL datasets from heart and blood tissues were analyzed using two-sample MR, Steiger filtering, and summary-data-based MR (SMR). Single-cell transcriptomic data (GSE145154) from DCM and control hearts were processed for cellular annotation, communication, and pseudo-time analysis.

**Results:**

MR and co-localization identified IMPA1 and ITIH4 as protective candidates for DCM, with consistent evidence across cardiac and blood tissues (PPH4 > 0.75). SMR and HEIDI tests confirmed shared causal variants between protein expression and DCM. Single-cell analysis revealed reduced IMPA1 expression in activated fibroblasts of DCM hearts, implicating inositol metabolism dysregulation in fibrosis. ITIH4 showed associations with metabolic traits but no adverse cardiac effects. Fibroblast subpopulations exhibited altered communication and differentiation trajectories in DCM, highlighting their role in disease progression.

**Conclusion:**

This multi-omics study prioritizes IMPA1 and ITIH4 as transcriptomic candidates with suggestive causal associations to DCM, linking inositol signaling and extracellular matrix stability to disease mechanisms. These findings underscore the potential of integrating genomics and single-cell transcriptomics to accelerate drug discovery in cardiovascular diseases.

**Supplementary Information:**

The online version contains supplementary material available at 10.1186/s41065-025-00539-9.

## Introduction

Dilated cardiomyopathy (DCM) is a myocardial disorder characterized by unilateral or bilateral ventricular dilatation and impaired systolic function [1, 2]. It is a leading cause of heart failure and sudden cardiac death worldwide, with an incidence of approximately 1 in 250 individuals and a prevalence in males about 1.5 times higher than that in females [3, 4]. Despite recent progress in symptom-focused pharmacologic therapies, including β-blockers, angiotensin-converting enzyme inhibitors, and mineralocorticoid receptor antagonists, the identification of drug targets responsible for DCM progression remains elusive [5]. The persistent lack of targeted interventions underscores a critical imperative to identify novel molecular pathways or mechanistic targets for therapeutic development [6, 7].

Recent developments in genome-wide association studies (GWAS) have facilitated the identification of genetic loci linked to cardiovascular diseases (CVDs) [8]. However, many disease-associated singl-nucleotide polymorphisms (SNPs) influence gene regulation indirectly rather than altering protein function, which complicates the identification of potential transcriptomic candidate genes relevant to disease etiology [9]. The “druggable genome” encompasses the subset of genes encoding proteins amenable to pharmacological modulation [10]. Previous studies have shown that drug development programs targeting validated genes have demonstrated a high success rate [11]. Therefore, the strategy of combining drugable genomes with GWAS is crucial for advancing precision therapy in DCM.

Mendelian randomization (MR) leverages SNPs as instrumental variables (IVs), exploiting the fact that genetic variation is fixed at conception and largely insulated from environmental and behavioral influences [12]. This characteristic enables MR to capture the lifelong effects of genetically mediated exposures on disease risk, establishing it as a powerful tool for the identification and validation of candidate genes. Meanwhile, advances in single-cell sequencing technologies and the convergence of diverse omics platforms, including proteomic, transcriptomic, expression quantitative trait loci (eQTL), and GWAS data, have enabled high-resolution mapping of gene regulatory networks implicated in DCM. In this study, we systematically assess the causal influence of the druggable genes on DCM by integrating eQTL, protein quantitative trait loci (pQTL), and single-cell transcriptomic data [13]. The integrative multi-omics approach seeks to deepen understanding of DCM pathogenesis and to accelerate the identification of innovative candidate genes.

## Method

### Study design

**Fig. 1:**
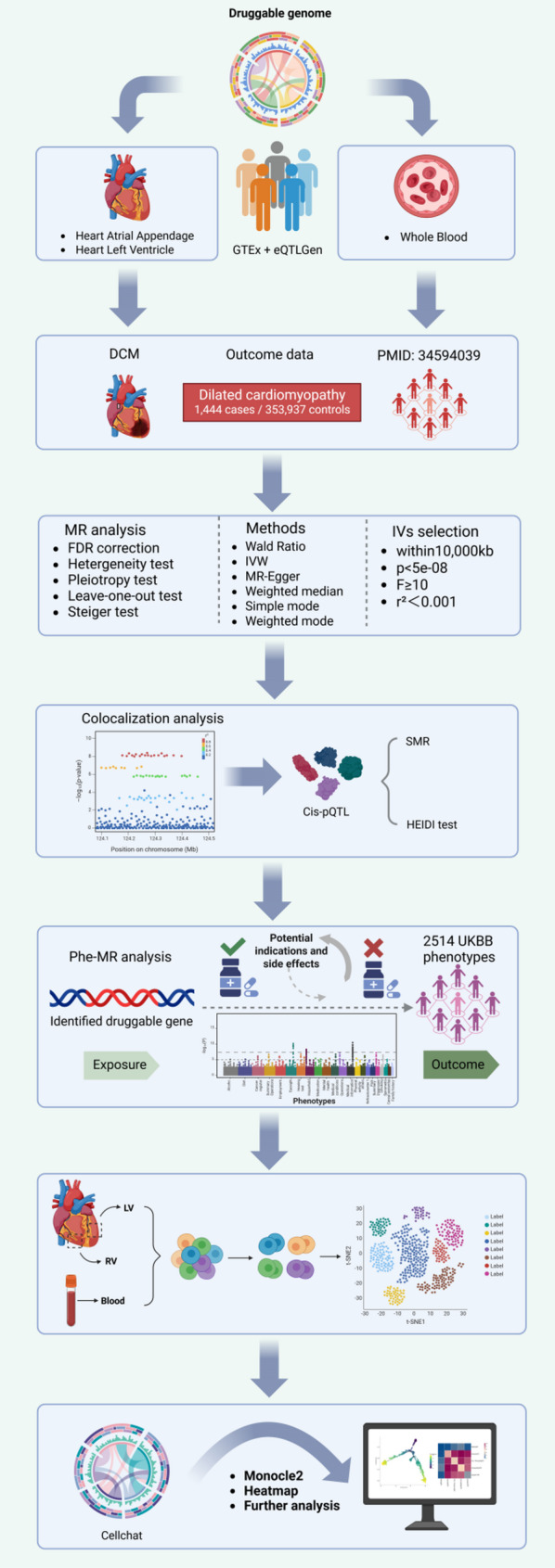
Study design overview

The research design, following the Strengthening the Reporting of Observational Studies in Epidemiology Using MR (STROBE-MR) guidelines (Supplementary Note 1), is outlined in the flowchart in Fig. 1. Analyses were conducted using aggregated data from multiple independent cohorts. Ethical approvals and informed consent were obtained by the original studies that generated the primary data.

We first curated a set of 5,900 druggable genes from three pharmacological databases. To investigate their potential causal roles in DCM, we conducted two-sample MR analyses utilizing eQTL derived from heart and whole blood tissues. Sensitivity analyses, including Steiger filtering and Bayesian co-localization, were employed to validate the robustness of causal inference. To further refine candidate genes, we integrated pQTL data from three independent sources, applying SMR and HEIDI outlier tests to identify pleiotropy-free associations. We next assessed the broader phenotypic consequences of these genes via Phe-MR to detect potential therapeutic indications and adverse effects. At last, we used the DCM single-cell sequencing dataset GSE145154 to explore the expression of the druggable gene in single cells.

### Identification of druggable genes

Druggable genes analyzed in this study were sourced from the Drug–Gene Interaction Database (DGIdb v4.2.0; February 2022 release, https://www.dgidb.org/downloads) and from previously published curated datasets (Table S1) [9, 14, 15]. DGIdb consolidates gene–drug interaction data from diverse publications, databases, and online resources. We retrieved the “category data” from DGIdb, encompassing genes classified as druggable and linked to the Entrez Gene database [15].

### eQTL and pQTL datasets

Given that cis-eQTLs are located proximal to their target genes and exhibit more direct functional effects compared with trans-eQTLs, cis-eQTLs were selected as IVs for the genes analyzed in this study [16]. Blood cis-eQTL GWAS summary statistics were obtained from the eQTLGen Consortium (https://eqtlgen.org/) and the Genotype-Tissue Expression (GTEx) project V8 (https://gtexportal.org/home/datasets)^17^. The eQTLGen dataset includes cis-eQTL summary statistics for 16,989 genes derived from 31,684 blood samples of European ancestry, whereas the GTEx dataset provides corresponding data from 670 whole blood tissue samples [17]. In addition, cis-eQTL GWAS summary statistics for two cardiac tissues, atrial appendage (*n* = 372) and left ventricle (*n* = 386), were extracted via the GTEx consortium (Table S1). For blood cis-pQTLs, GWAS summary statistics were sourced from three large-scale proteomic studies: the Fenland study [18] the UKB-PPP study [19] and the deCODE study [16]. Further methodological details are available in the original publications.

### DCM dataset

GWAS summary statistics for DCM were obtained from Jurgens et al., comprising 9,365 cases and 946,368 controls [20]. The dataset integrated six cohorts, including three clinically ascertained case-control studies and three population-based biobank datasets (FinnGen, UK Biobank, and Mass General Brigham Biobank) [20]. Clinical DCM cases were confirmed by imaging criteria, while biobank cases were defined using a strict non-ischemic DCM phenotype based on ICD-10 code I42.0, excluding individuals with ischemic heart disease [20]. This harmonized approach ensured high specificity in case definition and enabled robust meta-analysis across diverse cohorts.

### IVs selection

IVs used in MR were required to meet three core assumptions: (i) a strong link to the druggable genome; (ii) independence from confounding factors; and (iii) a specific effect on DCM mediated solely through the target gene, without influence from alternative biological pathways. In this study, cis-regions were defined as genomic intervals extending 1 Mb upstream and downstream of the coding sequence of each druggable genome. IVs were selected based on genome-wide significance (*P* < 5 × 10⁻⁸) and subjected to stringent filtering to ensure instrument strength and independence. Specifically, IVs with F-statistics < 10 or in linkage disequilibrium (r² >0.001 within a 10,000 kb window), as estimated from European-ancestry samples in the 1000 Genomes Project, were excluded. These criteria were applied to minimize the impact of weak IVs and horizontal pleiotropy, thereby improving the robustness of causal inference.

### MR and Steiger filtering analysis

SNPs related to the druggable genome were retrieved from the GWAS summary statistics for DCM, removing those with ambiguous strand orientation, such as moderate palindromic variants. In two-sample MR analyses, the causal effect of each druggable gene was estimated using the Wald ratio method when only a single IV was available. For genes with two or more IVs, causal inference was primarily performed using the inverse variance weighted (IVW) method [21]. When three or more IVs were present, the IVW estimates were further supported by four complementary MR methods: MR-Egger regression, weighted median, weighted mode, and simple mode. Multiple testing correction was applied using the false discovery rate (FDR) method, and a causal relationship was considered robust if the FDR-adjusted IVW *P*-value was below 0.05 and the direction of the effect (β) was consistent across all five MR methods. Horizontal pleiotropy was assessed using the MR-Egger intercept, while heterogeneity among instruments was evaluated via Cochran’s Q statistic within the IVW framework [22]. A random-effects model was applied when heterogeneity was significant (*P* < 0.05); otherwise, a fixed-effects model was used. To further validate the findings, we conducted leave-one-out sensitivity analyses and applied the MR-PRESSO method to identify and correct for outlier SNPs. To further address reverse causality, steiger filtering was conducted to minimize its potential impact. The TwoSampleMR package (v0.6.6) in R (version 4.4.1) was employed to perform all analyses.

### Colocalization analysis

Bayesian co-localization analysis was conducted using the COLOC R package (v5.2.3) to determine whether druggable genes and DCM are driven by a shared causal variant. This approach estimates the posterior probabilities (PP) for five scenarios: PPH0, representing the absence of association with either trait; PPH1, denoting association with gene expression alone; PPH2, association with DCM only; PPH3, association with both traits driven by distinct causal variants; and PPH4, association with both traits driven by a shared causal variant [22]. For each analysis, we extracted SNPs located within ± 100 kilobases of the druggable gene region. A gene was considered to exhibit evidence of co-localization with DCM if the PPH4 exceeded 0.75. This threshold was chosen to enhance the stringency and reliability of the inference.

### SMR analysis and HEIDI test

Summary data–based MR (SMR) was applied to assess the potential causal relationship between protein expression and DCM. In this approach, the SNP exhibiting the strongest association (lowest *P*-value) within the cis-region is selected as the IV, and the Heterogeneity in Dependent Instruments (HEIDI) test is used to determine whether the cis-pQTL and DCM associations are attributable to a shared causal variant [23]. SNPs located within a ± 500-kilobase window of each protein-coding gene were included in the analysis. Cis-pQTL summary statistics were derived from three extensive genome-wide proteomic cohorts (Table S1) and analyzed using the SMR software (version 1.3.1). A HEIDI *P*-value greater than 0.05 was considered indicative of a shared genetic signal between protein expression and DCM, supporting a putative causal relationship.

### Phenome-wide MR and tissue-specific expression of druggable genes

To further explore the clinical relevance of druggable genes identified through MR, we conducted a phenotype-wide MR (Phe-MR) analysis. In this framework, druggable genes served as exposure variables, and genome-wide summary statistics for a wide range of disease phenotypes from the UK Biobank (*n* ≤ 463,010) were used as outcomes. This analysis aimed to identify potential therapeutic indications, as well as adverse effects, associated with the modulation of the identified targets. Statistical significance was assessed using the Bonferroni correction to account for multiple testing, with *P*-values below the corrected threshold considered significant.

### Quality control and cellular annotation

The single-cell RNA sequencing dataset for DCM was obtained from the Gene Expression Omnibus (GEO) database (https://www.ncbi.nlm.nih.gov/gds, GSE145154), including 5 Control samples and 10 DCM samples [24]. Strict quality control was performed to ensure the quality of the single-cell data. When filtering cells, we excluded cells with gene expression numbers less than 200 or more than 98% of the gene expression of the sample. Meanwhile, we also controlled for the number of RNA molecules in a single cell (nCountRNA range: min = 0, max = 98% number of RNA molecules per sample). In addition, mitochondrial content is an important reference factor, and we chose the threshold closest to filtering 5% of cells to exclude cells with excessive mitochondrial gene expression, thus improving data quality and providing more reliable cell samples for subsequent analyses. Finally, we normalised the data and selected up to 2,000 highly variable genes [24].

According to Seurat’s standard procedure, principal component analysis was used to downscale the data, and the top 20 principal components were selected for further nonlinear downscaling using UMAP [24]. A clustering resolution of 0.8 was selected for mechanical clustering of cells, which was automatically annotated based on the Cell marker-based database and manually managed to further refine the annotation according to the available literature. Based on the results of cellular annotation, we analysed the expression and distribution of potential DCM targets in different types. In addition, we used the ratio of observed to expected cell numbers (Ro/e) to quantify the degree of tissue enrichment to determine whether cell populations have different preferences between tissues. This method assesses whether the distribution of cell subpopulations in tissues significantly deviates from random expectations by means of a chi-square test, and can effectively quantify the tissue preference of cell subpopulations [25].

### Cellular communication and pseudo-time analysis

To further explore the interactions between cells in the DCM microenvironment, we performed cellular communication analysis using CellPhoneDB, a database containing ligands, receptors, and their interactions. Currently, CellPhoneDB has a total of 1,419 proteins involved in 2,911 interacting gene pairs and is able to infer intercellular communication relationships based on single-cell gene expression data [26]. In addition, to clarify the relationship between the differentiated development of key cell subpopulations and DCM, we performed a pseudo-time analysis using monocle2^27, 28^.

## Results

### Druggable genome

We compiled a comprehensive reference set of 5,900 unique druggable genes by integrating data from three sources: 3,953 genes from the DGIdb, 4,463 genes from Finan et al. [9] and 269 literature-supported genes from Schneider et al. [14]. Gene symbols were standardized using Human Genome Organisation Gene Nomenclature Committee annotations. This harmonized gene set formed the basis for the subsequent cis-eQTL, cis-pQTL, and MR analyses (Table S2).

### Candidate druggable genes for DCM

We selected IVs from four distinct eQTL datasets: atrial appendage, left ventricle, eQTLGen, and whole blood. In the subsequent two-sample MR analyses, we observed suggestive evidence of causality across all four datasets (Table S3). An FDR-corrected *P*-value below 0.05 was defined as indicative of a significant association. For DCM, we identified 27, 24, 18, and 13 significantly associated druggable genes in the atrial appendage, left ventricle, eQTLGen, and whole blood datasets, respectively. To ensure analytical rigor, genes that demonstrated a significant association in at least two eQTL datasets were retained for further Bayesian co-localization analysis.

**Fig. 2:**
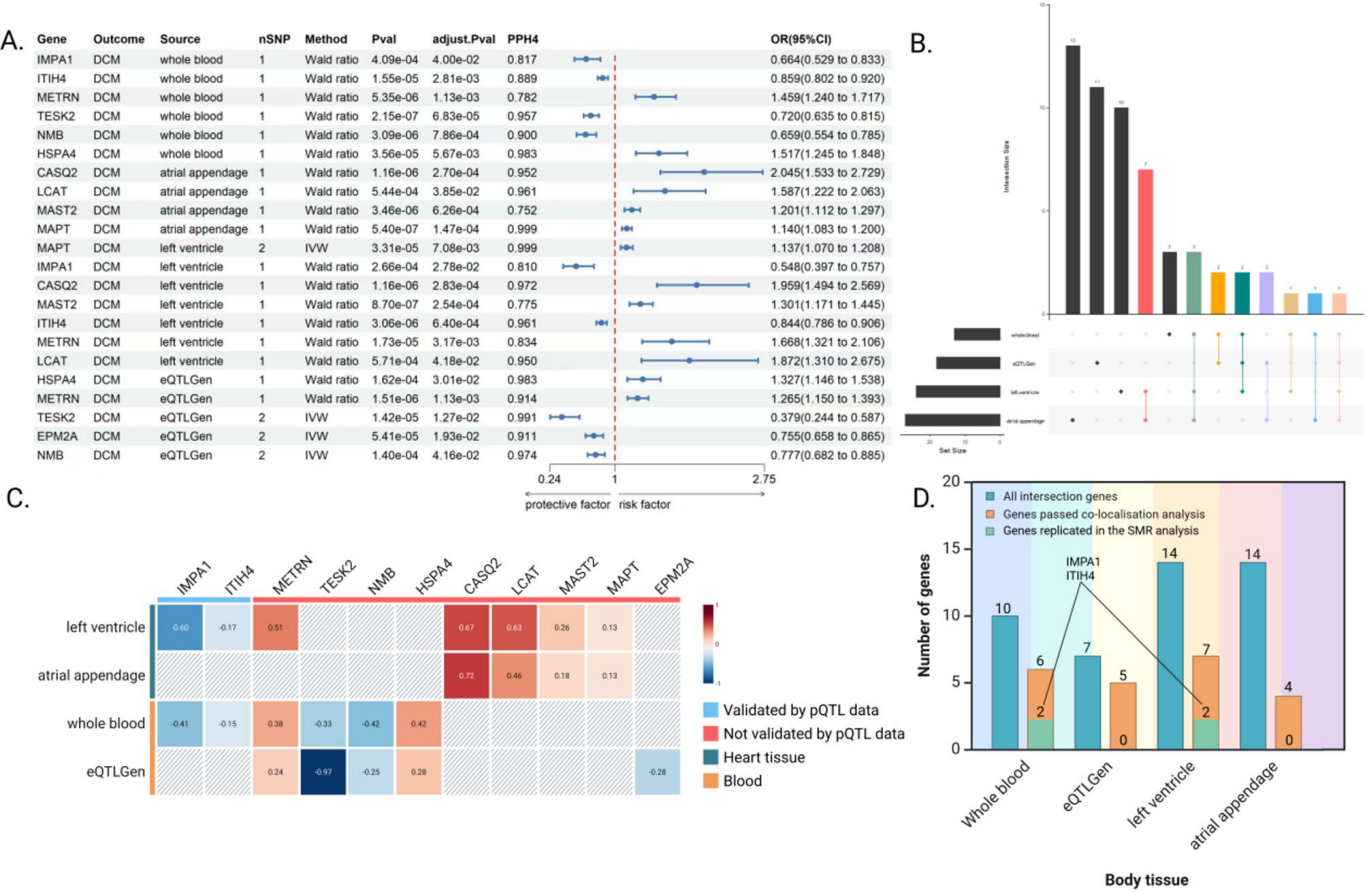
DCM potential drug targets selection process and results. **A.** Presence of druggable genes in DCM and PPH4 > 75%. **B.** For each tissue, genes were extracted by co-localization analysis. **C.** Correlations between localizable drug genes and DCM in different tissues, values indicate the degree of beta, red indicates a risk effect, and blue suggests a protective effect. **D.** The number of genes in each tissue and the genes that can be reproduced in the SMR were found by co-localisation analysis

Co-localization results revealed that 11 genes exhibited strong evidence of sharing a causal variant with DCM (PPH4 > 0.75). To visualize the findings from both MR and co-localization analyses, we generated forest plots (Fig. 2A). Further validation was conducted using SMR and the HEIDI test, based on cis-pQTL data.

Among the 11 candidate genes, IMPA1 and ITIH4 met the validation criteria. IMPA1 showed strong co-localization in whole blood (PPH4 = 0.817, OR = 0.66, 95% CI: 0.53–0.83, *P* = 4.09 × 10⁻⁴) and left ventricle (PPH4 = 0.810, OR = 0.55, 95% CI: 0.40–0.76, *P* = 2.66 × 10⁻⁴), while ITIH4 demonstrated even stronger evidence in whole blood (PPH4 = 0.889, OR = 0.86, 95% CI: 0.80–0.92, *P* = 1.55 × 10⁻⁵) and left ventricle (PPH4 = 0.961, OR = 0.84, 95% CI: 0.79–0.91, *P* = 3.06 × 10⁻⁶) (Fig. 2A). Both genes achieved significance in SMR analysis (*P* < 0.05) and passed the HEIDI test (*P* > 0.05), suggesting a shared causal variant between protein expression and DCM. Based on these findings, IMPA1 and ITIH4 emerge as promising drug targets for the treatment of DCM (Fig. 2B and D).

### Phe-MR analysis of drug candidate genes for DCM

Phe-MR analysis was conducted using summary statistics encompassing 2,514 diseases and traits from the UK Biobank (Table S6). IVs comprised four SNPs located in IMPA1 and ITIH4 (Table S7). Bonferroni correction was applied to account for multiple testing. The results revealed no significant associations between IMPA1 and any additional phenotypes. In contrast, ITIH4 was significantly associated with nervousness or high-strung behavior, decreased standing height, and a reduced risk of increased body mass index (Table S8). Manhattan plots were generated to visualize the Phe-MR results (Figures S1–S4).

### Quality control and cellular annotation

**Fig. 3:**
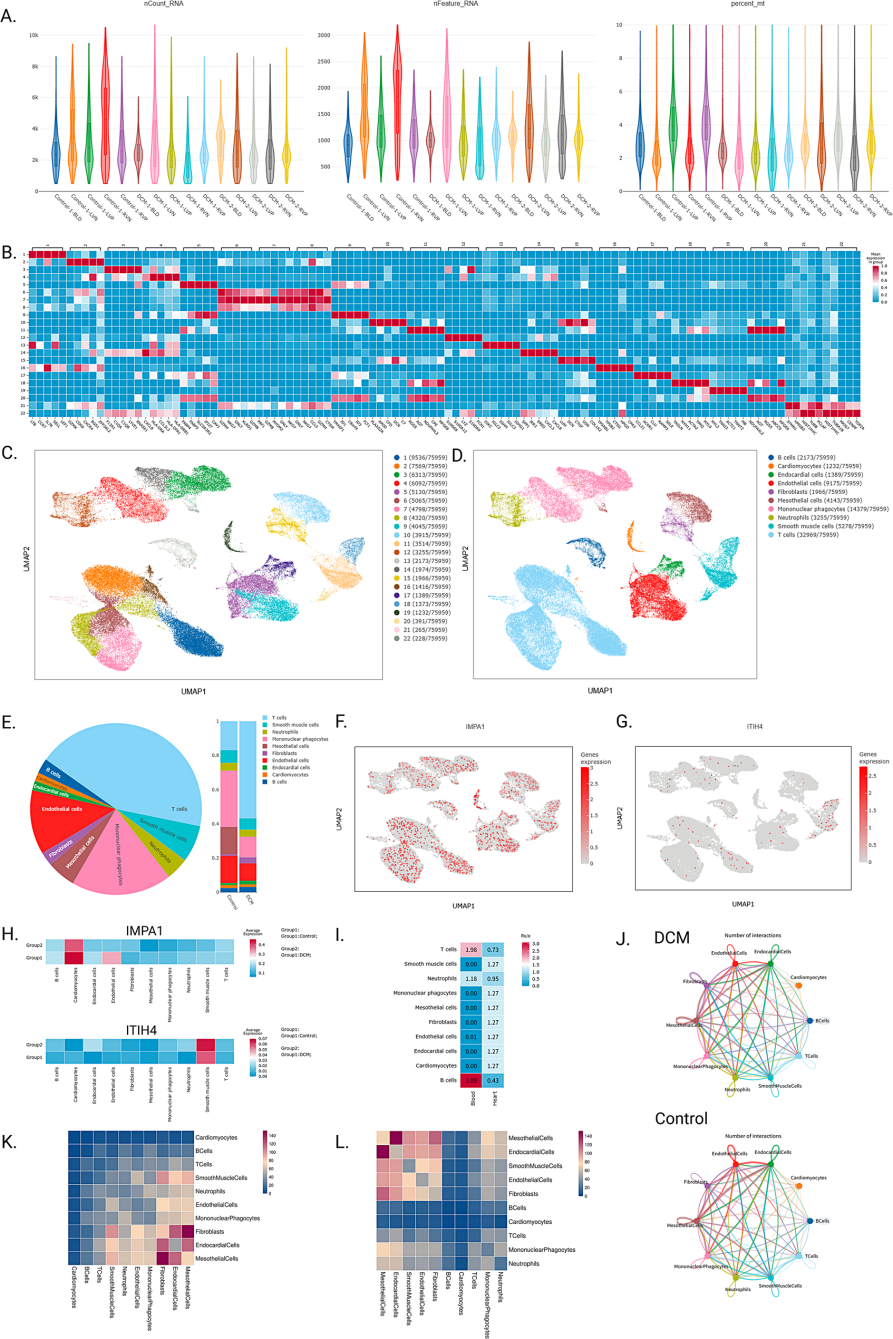
Results of cell quality control and single-cell analysis. (**A**) Results of single-cell quality control (**B**) Heatmap of mean values based on marker genes **C-D**. Results of mechanical clustering and cell annotation based on a clustering resolution of 0.8 E. Overall cell occupancy and cell occupancy between the two groups **F-H**. Expression of IMPA1 and ITIH4 in different cellular subpopulations I. Ro/e analyses reveal the preference of different cellular subpopulations in the tissue **J-K** Results of cellular communication between the two groups

After quality control, we obtained 75,959 cells from 15 samples (Fig. 3C and Table S9). The cells were classified into 22 clusters based on a clustering resolution of 0.8 and cell annotation was performed based on marker genes, and a total of 10 cell types were identified, including B cells, Cardiomyocytes, Endocardial cells, Endothelial cells, Fibroblasts, Mesothelial cells Mononuclear phagocytes, Neutrophils, Smooth muscle cells and T cells (Fig. 3D). Figure 3E demonstrates the overall cell percentage and the cell percentage between the two groups. Analysis of the Ro/e values revealed that T and B cells were predominantly enriched in the blood. Neutrophils had Ro/e values of 1.18 and 0.95 in the blood and heart, whereas the remaining cellular subpopulations functioned predominantly in cardiac tissues (Fig. 3I).

Based on these results, we characterised the expression of potential drug targets of DCM in different cell subpopulations (Fig. 3F-H). We found that the expression of the IMPA1 gene was reduced in the Control group compared to the DCM group, which partially validated the previous results of the drug target MR. However, although our MR revealed a protective role for ITIH4 in DCM, it was not consistent with the current analysis by single-cell sequencing (Fig. 3H). In addition, the results of cellular communication revealed that fibroblasts play an important role in cellular communication between the two groups (Fig. 3J-L). Figure 3 shows the results of cell quality control and single-cell analysis.

### Cellular communication and pseudo-time analysis

**Fig. 4:**
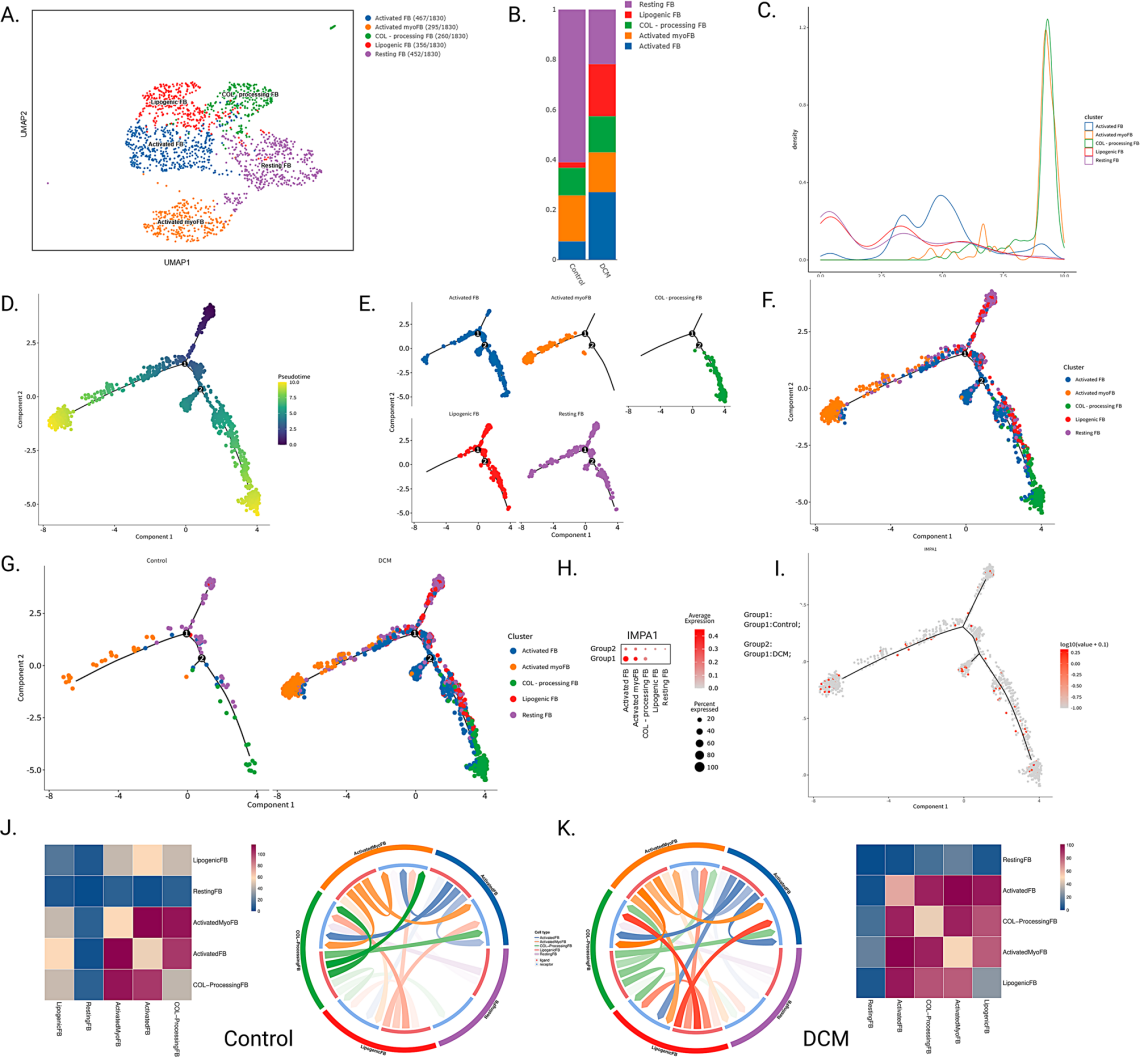
Results of cellular communication and pseudo-time analysis of fibroblast subpopulations. (**A**) Results of cellular annotation of fibroblast subpopulations using UMAP (**B**) Cell occupancy between the two groups (**C**) Density plots of cell occupancy over time progression **D-G**. Results of the pseudo-time analysis **H**. Expression of IMPA1 between different cellular subpopulations **I**. Expression of IMPA1 throughout the pseudo-time analysis **J-K**. Results of cellular communication between the two groups of fibroblast subpopulations

We further performed cellular annotation for fibroblasts, based on the marker gene provided by Rao et al. Since ITIH4 is hardly expressed in fibroblasts, we focused on IMPA1 for further analysis. We annotated fibroblasts into five cellular subpopulations of Activated FB, Activated myoFB, COL-processing FB, Lipogenic FB, and Resting FB (Fig. 4A) [29]. Figure 4B demonstrates the difference in the number of cells between Control and DCM groups, and we found that the number of Lipogenic FB cells and Activated FB cells was higher in the DCM group, while the number of Resting FB cells was higher in the Control group. Furthermore, we found that IMPA1 expression was reduced in the DCM group in Activated FB and Activated myoFB, suggesting a distinct role for these two fibroblast subpopulations in DCM progression (Fig. 4H).

To further clarify the differentiation and developmental relationships between the five cell subpopulations of fibroblasts, we performed a pseudo-time analysis using Monyuer2 (Fig. 4D). Figure 4D demonstrates the distribution of all cells in the proposed chronological analysis, with colours indicating pseudo-temporal progression. Notably, increased cell aggregation in certain regions was observed in the DCM group (e.g., Activated FB, Lipogenic FB, and COL-processing FB), and this difference in cell distribution pattern may reflect the underlying pathological features of myofibrosis in the centre of DCM (Fig. 4E-G). Expression of IMPA1 on the developmental trajectory of fibroblast subpopulation differentiation showed consistent expression of IMPA1 at all developmental stages (Fig. 4I). In addition, in the situation of DCM, we observed enhanced cellular communication between Lipogenic FB cells and Activated FB and COL-processing FB, which may reflect the role of lipid metabolism in DCM progression. Figure 4 shows the results of cellular communication and pseudo-time analysis of fibroblast subpopulations.

## Discussion

By integrating tissue-specific eQTL, pQTL, and single-cell transcriptomic data with MR and co-localization analyses, we identified IMPA1 and ITIH4 as candidate pharmacological targets for DCM. Both IMPA1 and ITIH4 exhibited protective associations in whole blood and left ventricular tissue. Phe-MR further implicated these genes in additional therapeutic contexts. Single-cell transcriptomic analysis revealed reduced IMPA1 expression in a subset of activated fibroblasts within DCM hearts, implicating impaired myoinositol metabolism in fibrotic remodeling. Collectively, these multi-omics findings nominate IMPA1 and ITIH4 as transcriptomic candidates with suggestive causal associations to DCM and provide mechanistic insights into disease pathogenesis.

IMPA1 (inositol monophosphatase 1), a pivotal enzyme in inositol phosphate metabolism, regulates intracellular signaling and energy homeostasis by hydrolyzing inositol monophosphates to sustain inositol recycling [30]. Although dysregulated inositol pathways have been implicated in cardiac hypertrophy and fibrosis, the specific contribution of IMPA1 to DCM has remained undefined [31]. In our investigation, single-cell RNA sequencing revealed reduced IMPA1 expression in activated fibroblasts from DCM hearts, suggesting a potential role in restraining fibrotic activation. This observation is consistent with recent evidence that inositol signaling modulates fibroblast-to-myofibroblast transition via TGF-β signaling [32]. Mechanistically, loss of IMPA1 may impair inositol recycling, thereby promoting mitochondrial dysfunction and oxidative stress within cardiac muscle cells, an interpretation supported by proteomic studies linking inositol metabolism to cardiac energetics [33].

ITIH4 (α-trypsin interstitial inhibitor heavy chain 4) is a hyaluronan-binding protein involved in extracellular matrix (ECM) stabilization and inflammatory regulation, but has not previously been considered causally related to DCM [34]. Although early proteomics studies linked ECM disruption to DCM progression, the functional contribution of ITIH4 remains unresolved [35, 36]. In our research, MR and co-localization analyses identified reduced ITIH4 expression as a risk factor for DCM, possibly through increased myocardial stiffness and ventricular dilatation. Single-cell RNA sequencing localized ITIH4 expression to smooth muscle cells, suggesting a role for ITIH4 in maintaining vascular structure and ECM homeostasis. These findings are in contrast to previous candidate gene studies, which failed to prioritize ITIH4, possibly because of the multidirectional association of ITIH4 with metabolic phenotypes, as emphasized by our Phe-MR analysis.

As far as we know, this is the first MR research to systematically discover candidate genes for DCM by integrating multiple genomic datasets. We report three key contributions. First, we conducted a comprehensive analysis of potential drug target genes by leveraging a range of eQTL datasets. Second, we substantiated these findings utilizing pQTL datasets and applied SMR alongside HEIDI testing, thereby enhancing the robustness and validity of the associations. Third, Phe-MR analyses revealed potential adverse effects and alternative indications associated with the identified DCM targets, offering critical insights for translational applications. This study combines the analysis of heart tissue and blood cells to reveal the expression of IMPA1 and ITIH4 in single cells.

This study has several limitations. First, the associations between IMPA1, ITIH4, and DCM were identified at the transcriptomic level; more experimental validation is needed to clarify the specific mechanism of action of ITIH4 and IMPA1 in fibroblasts. Second, genetic variants used as instruments for gene expression may influence multiple transcripts across different loci, introducing pleiotropy that can bias MR estimates. Third, due to the elongated shape and large size of cardiomyocytes, most cardiomyocytes can not be effectively captured using the 10x platform, and therefore, future studies need snRNAseq data for further analysis. Lastly, as our analyses were based exclusively on individuals of European ancestry, the translatability of these findings to other populations warrants further investigation in more diverse genetic cohorts.

## Conclusion

In summary, this investigation integrates single-cell transcriptomics and Mendelian randomization to identify IMPA1 and ITIH4 as transcriptomic candidates with suggestive causal associations to DCM. These findings advance a mechanistic framework for precision drug development in DCM, underscoring the translational power of genomic approaches in cardiovascular medicine.

## Supplementary Information

Below is the link to the electronic supplementary material.

Supplementary Material 1

Supplementary Material 2

Supplementary Material 3

## Data Availability

No datasets were generated or analysed during the current study.

## Abbreviations

MR: Mendelian randomization
SMR: Summary-data-based mendelian randomization
DCM: Dilated Cardiomyopathy
CVDs: Cardiovascular diseases
eQTL: Expression quantitative trait loci
pQTL: Protein quantitative trait loci
GEO: Gene Expression Omnibus
Phe-MR: Phenome‐wide Mendelian randomization
GWAS: Genome-wide association studies
DGIdb: Drug–Gene Interaction Database
IVs: Instrumental variables
FDR: False discovery rate
GTEx: Genotype-Tissue Expression
IVW: Inverse variance–weighted
LD: Linkage disequilibrium
SNPs: Single nucleotide polymorphisms
PP: Posterior probabilities
IMPA1: Inositol monophosphatase 1
ITIH4: α-trypsin interstitial inhibitor heavy chain 4
ECM: Extracellular matrix

## References

[1] Hershberger RE, Hedges DJ, Morales A. Dilated cardiomyopathy: the complexity of a diverse genetic architecture. Nat Rev Cardiol. 2013;10:531–47. 10.1038/nrcardio.2013.105. 23900355 10.1038/nrcardio.2013.105

[2] McNally EM, Mestroni L. Dilated cardiomyopathy: genetic determinants and mechanisms. Circ Res. 2017;121:731–48. 10.1161/CIRCRESAHA.116.309396. 28912180 10.1161/CIRCRESAHA.116.309396 PMC5626020

[3] Fatkin D, Calkins H, Elliott P, James CA, Peters S, Kovacic JC. Contemporary and future approaches to precision medicine in inherited cardiomyopathies: JACC focus seminar 3/5. J Am Coll Cardiol. 2021;77:2551–72. 10.1016/j.jacc.2020.12.072. 34016267 10.1016/j.jacc.2020.12.072

[4] Charron P, Elliott PM, Gimeno JR, Caforio A, Kaski JP, Tavazzi L, Tendera M, Maupain C, Laroche C, Rubis P, et al. The cardiomyopathy registry of the eurobservational research programme of the European society of cardiology: baseline data and contemporary management of adult patients with cardiomyopathies. Eur Heart J. 2018;39:1784–93. 10.1093/eurheartj/ehx819. 29378019 10.1093/eurheartj/ehx819

[5] Marian AJ, Braunwald E. Hypertrophic cardiomyopathy: genetics, pathogenesis, clinical manifestations, diagnosis, and therapy. Circ Res. 2017;121:749–70. 10.1161/CIRCRESAHA.117.311059. 28912181 10.1161/CIRCRESAHA.117.311059 PMC5654557

[6] Nelson MR, Tipney H, Painter JL, Shen J, Nicoletti P, Shen Y, Floratos A, Sham PC, Li MJ, Wang J, et al. The support of human genetic evidence for approved drug indications. Nat Genet. 2015;47:856–60. 10.1038/ng.3314. 26121088 10.1038/ng.3314

[7] Hopkins AL, Groom CR. The druggable genome. Nat Rev Drug Discov. 2002;1:727–30. 10.1038/nrd892. 12209152 10.1038/nrd892

[8] Liu R, Sun C, Li J, Yang G, Xu K, Hu J, Meng C, Xia X, Li Y, Liu Y. Identifying potential drug targets in coronary atherosclerosis: insights from the druggable genome and Mendelian randomization. Cardiovasc Drugs Ther. 2025. 10.1007/s10557-025-07694-1. 40214710 10.1007/s10557-025-07694-1

[9] Finan C, Gaulton A, Kruger FA, Lumbers RT, Shah T, Engmann J, Galver L, Kelley R, Karlsson A, Santos R, et al. The druggable genome and support for target identification and validation in drug development. Sci Transl Med. 2017;9. 10.1126/scitranslmed.aag1166. 10.1126/scitranslmed.aag1166 PMC6321762 28356508

[10] Ochoa D, Karim M, Ghoussaini M, Hulcoop DG, McDonagh EM, Dunham I. Human genetics evidence supports two-thirds of the 2021 FDA-approved drugs. Nat Rev Drug Discov. 2022;21:551. 10.1038/d41573-022-00120-3. 35804044 10.1038/d41573-022-00120-3

[11] Liu J, Zeng D, Wang Y, Deng F, Wu S, Deng Z. Identification of druggable targets in acute kidney injury by proteome- and transcriptome-wide Mendelian randomization and bioinformatics analysis. Biol Direct. 2025;20:38. 10.1186/s13062-025-00631-0. 40148878 10.1186/s13062-025-00631-0 PMC11951703

[12] Liu R, Fan W, Hu J, Xu K, Huang Z, Liu Y, Sun C. The mediating role of thyroid-related hormones between thyroid dysfunction diseases and osteoporosis: a mediation Mendelian randomization study. Sci Rep. 2025;15:4121. 10.1038/s41598-025-88412-7. 39901040 10.1038/s41598-025-88412-7 PMC11791035

[13] Hao RH, Zhang TP, Jiang F, Liu JH, Dong SS, Li M, Guo Y, Yang TL. Revealing brain cell-stratified causality through dissecting causal variants according to their cell-type-specific effects on gene expression. Nat Commun. 2024;15:4890. 10.1038/s41467-024-49263-4. 38849352 10.1038/s41467-024-49263-4 PMC11161590

[14] Schneider M, Radoux CJ, Hercules A, Ochoa D, Dunham I, Zalmas LP, Hessler G, Ruf S, Shanmugasundaram V, Hann MM, et al. The protactable genome. Nat Rev Drug Discov. 2021;20:789–97. 10.1038/s41573-021-00245-x. 34285415 10.1038/s41573-021-00245-x

[15] Freshour SL, Kiwala S, Cotto KC, Coffman AC, McMichael JF, Song JJ, Griffith M, Griffith OL, Wagner AH. Integration of the Drug-Gene interaction database (DGIdb 4.0) with open crowdsource efforts. Nucleic Acids Res. 2021;49:D1144–51. 10.1093/nar/gkaa1084. 33237278 10.1093/nar/gkaa1084 PMC7778926

[16] Ferkingstad E, Sulem P, Atlason BA, Sveinbjornsson G, Magnusson MI, Styrmisdottir EL, Gunnarsdottir K, Helgason A, Oddsson A, Halldorsson BV, et al. Large-scale integration of the plasma proteome with genetics and disease. Nat Genet. 2021;53:1712–21. 10.1038/s41588-021-00978-w. 34857953 10.1038/s41588-021-00978-w

[17] Battle A, Brown CD, Engelhardt BE, Montgomery SB. Genetic effects on gene expression across human tissues. Nature. 2017;550:204–13. 10.1038/nature24277. 29022597 10.1038/nature24277 PMC5776756

[18] Pietzner M, Wheeler E, Carrasco-Zanini J, Cortes A, Koprulu M, Worheide MA, Oerton E, Cook J, Stewart ID, Kerrison ND, et al. Mapping the proteo-genomic convergence of human diseases. Science. 2021;374:eabj1541. 10.1126/science.abj1541. 34648354 10.1126/science.abj1541 PMC9904207

[19] Sun BB, Chiou J, Traylor M, Benner C, Hsu YH, Richardson TG, Surendran P, Mahajan A, Robins C, Vasquez-Grinnell SG, et al. Plasma proteomic associations with genetics and health in the UK biobank. Nature. 2023;622:329–38. 10.1038/s41586-023-06592-6. 37794186 10.1038/s41586-023-06592-6 PMC10567551

[20] Jurgens SJ, Ramo JT, Kramarenko DR, Wijdeveld L, Haas J, Chaffin MD, Garnier S, Gaziano L, Weng LC, Lipov A, et al. Genome-wide association study reveals mechanisms underlying dilated cardiomyopathy and myocardial resilience. Nat Genet. 2024;56:2636–45. 10.1038/s41588-024-01975-5. 39572784 10.1038/s41588-024-01975-5 PMC11631763

[21] Hartwig FP, Davey SG, Bowden J. Robust inference in summary data Mendelian randomization via the zero modal Pleiotropy assumption. Int J Epidemiol. 2017;46:1985–98. 10.1093/ije/dyx102. 29040600 10.1093/ije/dyx102 PMC5837715

[22] Giambartolomei C, Vukcevic D, Schadt EE, Franke L, Hingorani AD, Wallace C, Plagnol V. Bayesian test for colocalisation between pairs of genetic association studies using summary statistics. PLoS Genet. 2014;10:e1004383. 10.1371/journal.pgen.1004383. 24830394 10.1371/journal.pgen.1004383 PMC4022491

[23] Wu Y, Zeng J, Zhang F, Zhu Z, Qi T, Zheng Z, Lloyd-Jones LR, Marioni RE, Martin NG, Montgomery GW, et al. Integrative analysis of omics summary data reveals putative mechanisms underlying complex traits. Nat Commun. 2018;9:918. 10.1038/s41467-018-03371-0. 29500431 10.1038/s41467-018-03371-0 PMC5834629

[24] Butler A, Hoffman P, Smibert P, Papalexi E, Satija R. Integrating single-cell transcriptomic data across different conditions, technologies, and species. Nat Biotechnol. 2018;36:411–20. 10.1038/nbt.4096. 29608179 10.1038/nbt.4096 PMC6700744

[25] Zhang L, Yu X, Zheng L, Zhang Y, Li Y, Fang Q, Gao R, Kang B, Zhang Q, Huang JY, et al. Lineage tracking reveals dynamic relationships of T cells in colorectal cancer. Nature. 2018;564:268–72. 10.1038/s41586-018-0694-x. 30479382 10.1038/s41586-018-0694-x

[26] Jin S, Guerrero-Juarez CF, Zhang L, Chang I, Ramos R, Kuan CH, Myung P, Plikus MV, Nie Q. Inference and analysis of cell-cell communication using cellchat. Nat Commun. 2021;12:1088. 10.1038/s41467-021-21246-9. 33597522 10.1038/s41467-021-21246-9 PMC7889871

[27] Fischer DS, Fiedler AK, Kernfeld EM, Genga R, Bastidas-Ponce A, Bakhti M, Lickert H, Hasenauer J, Maehr R, Theis FJ. Inferring population dynamics from single-cell RNA-sequencing time series data. Nat Biotechnol. 2019;37:461–8. 10.1038/s41587-019-0088-0. 30936567 10.1038/s41587-019-0088-0 PMC7397487

[28] Qiu X, Mao Q, Tang Y, Wang L, Chawla R, Pliner HA, Trapnell C. Reversed graph embedding resolves complex single-cell trajectories. Nat Methods. 2017;14:979–82. 10.1038/nmeth.4402. 28825705 10.1038/nmeth.4402 PMC5764547

[29] Rao M, Wang X, Guo G, Wang L, Chen S, Yin P, Chen K, Chen L, Zhang Z, Chen X, et al. Resolving the intertwining of inflammation and fibrosis in human heart failure at single-cell level. Basic Res Cardiol. 2021;116:55. 10.1007/s00395-021-00897-1. 34601654 10.1007/s00395-021-00897-1

[30] Yuk CM, Hong S, Kim D, Kim M, Jeong HW, Park SJ, Min H, Kim W, Lim J, Kim HD, et al. Inositol polyphosphate multikinase regulates Th1 and Th17 cell differentiation by controlling Akt-mTOR signaling. Cell Rep. 2025;44:115281. 10.1016/j.celrep.2025.115281. 39946233 10.1016/j.celrep.2025.115281

[31] Lee TW, Chung CC, Lee TI, Lin YK, Kao YH, Chen YJ. Fibroblast growth factor 23 stimulates cardiac fibroblast activity through phospholipase C-Mediated calcium signaling. Int J Mol Sci. 2021;23. 10.3390/ijms23010166. 10.3390/ijms23010166 PMC8745152 35008591

[32] Zhang Y, Chen W, Wang Y. STING is an essential regulator of heart inflammation and fibrosis in mice with pathological cardiac hypertrophy via Endoplasmic reticulum (ER) stress. Biomed Pharmacother. 2020;125:110022. 10.1016/j.biopha.2020.110022. 32106379 10.1016/j.biopha.2020.110022

[33] Lkhagva B, Lin YK, Chen YC, Cheng WL, Higa S, Kao YH, Chen YJ. ZFHX3 knockdown dysregulates mitochondrial adaptations to tachypacing in atrial myocytes through enhanced oxidative stress and calcium overload. Acta Physiol (Oxf). 2021;231:e13604. 10.1111/apha.13604. 33332716 10.1111/apha.13604

[34] Ravindran A, Holappa L, Niskanen H, Skovorodkin I, Kaisto S, Beter M, Kiema M, Selvarajan I, Nurminen V, Aavik E, et al. Translatome profiling reveals Itih4 as a novel smooth muscle cell-specific gene in atherosclerosis. Cardiovasc Res. 2024;120:869–82. 10.1093/cvr/cvae028. 38289873 10.1093/cvr/cvae028 PMC11218691

[35] Jaber WA, Maniu C, Krysiak J, Shapiro BP, Meyer DM, Linke WA, Redfield MM. Titin isoforms, extracellular matrix, and global chamber remodeling in experimental dilated cardiomyopathy: functional implications and mechanistic insight. Circ Heart Fail. 2008;1:192–9. 10.1161/CIRCHEARTFAILURE.108.768465. 19808289 10.1161/CIRCHEARTFAILURE.108.768465 PMC2779561

[36] Collum SD, Chen NY, Hernandez AM, Hanmandlu A, Sweeney H, Mertens T, Weng T, Luo F, Molina JG, Davies J, et al. Inhibition of hyaluronan synthesis attenuates pulmonary hypertension associated with lung fibrosis. Br J Pharmacol. 2017;174:3284–301. 10.1111/bph.13947. 28688167 10.1111/bph.13947 PMC5595757

