# Supplementary material for: Integrating single-cell RNA sequencing and Mendelian randomization analysis to identify potential drug targets for dilated cardiomyopathy
Source: Hereditas. 2025 Oct 16;162:211. doi: 10.1186/s41065-025-00539-9 (PMC12532413; doi:10.1186/s41065-025-00539-9)
Supplement: Supplementary file 1 — Supplementary Material 1 [file 41065_2025_539_MOESM1_ESM.docx]

**Integrating Single-Cell RNA Sequencing and Mendelian Randomization Analysis to Identify Potential Drug Targets for Dilated Cardiomyopathy**

**
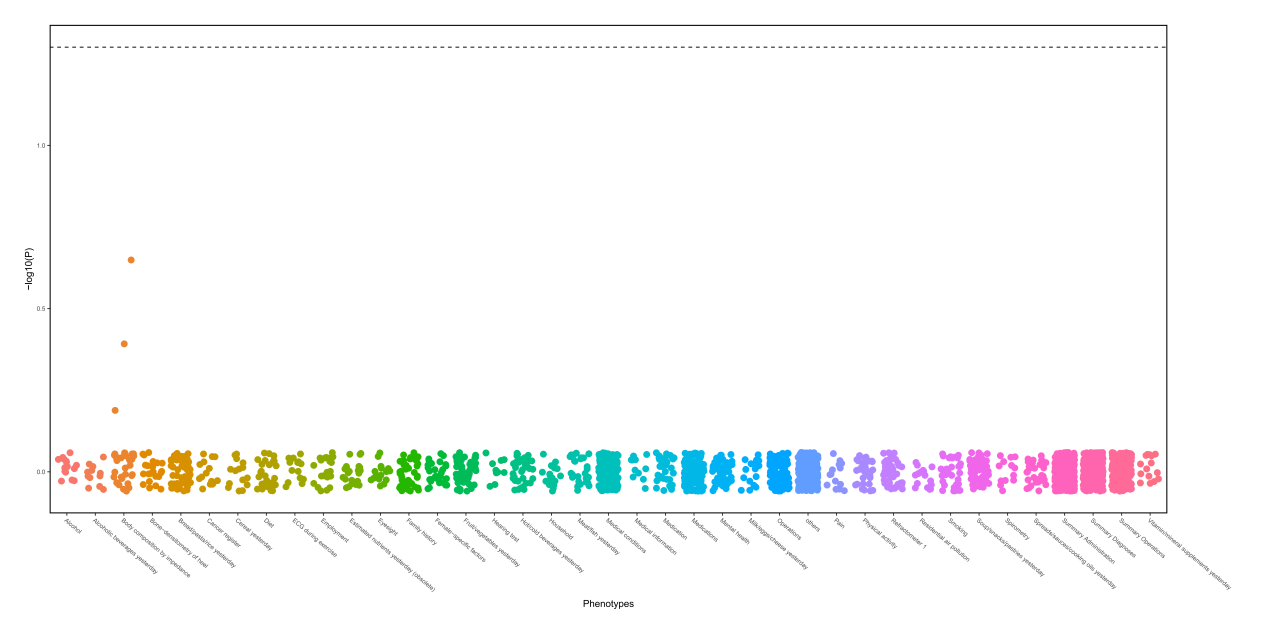
**

**Figure S1**

Phe-MR results, IMPA1 (left ventricle) as exposure, 2514 phenotypes from UKBB GWAS as outcome, dotted lines represent Bonferrion correction thresholds, specific positive results can be seen in Table S7.


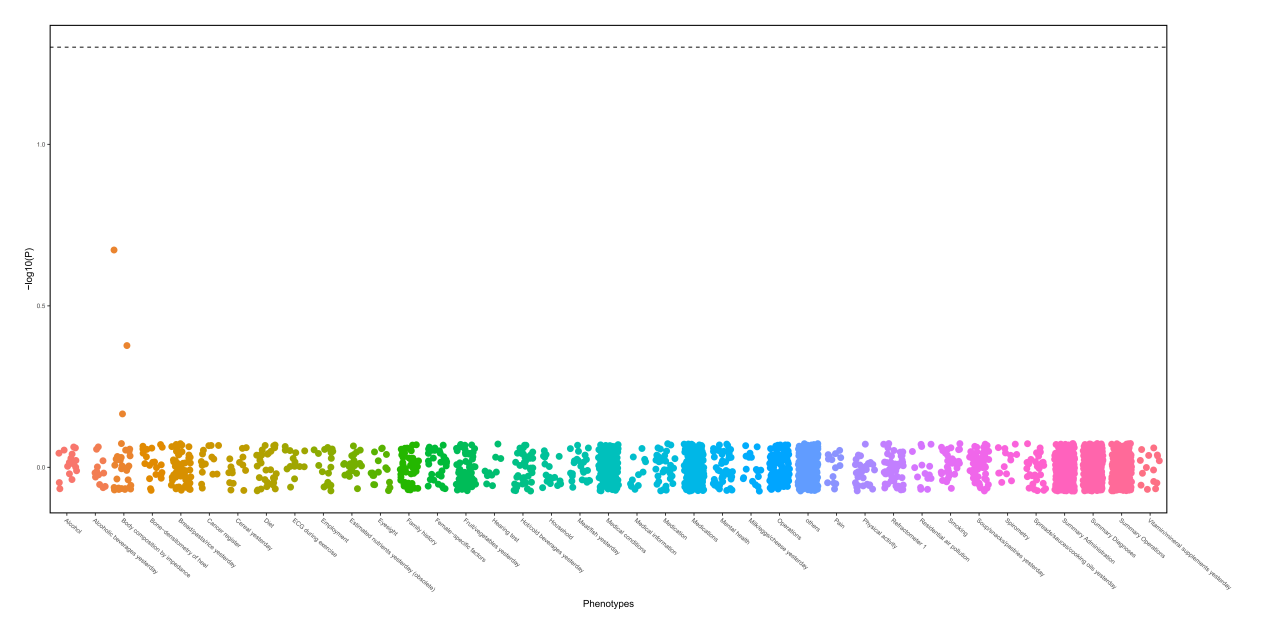


**Figure S2**

Phe-MR results, IMPA1 (whole blood) as exposure, 2514 phenotypes from UKBB GWAS as outcome, dotted lines represent Bonferrion correction thresholds, specific positive results can be seen in Table S7.


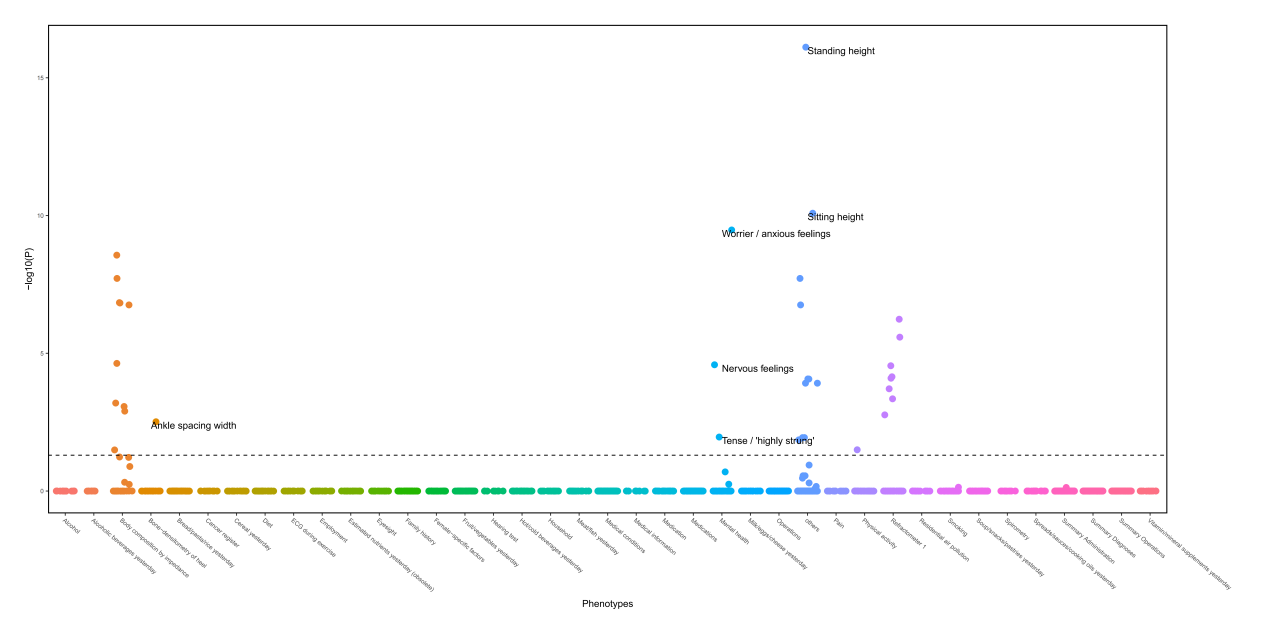


**Figure S3**

Phe-MR results, ITIH4 (left ventricle) as exposure, 2514 phenotypes from UKBB GWAS as outcome, dotted lines represent Bonferrion correction thresholds, specific positive results can be seen in Table S13.


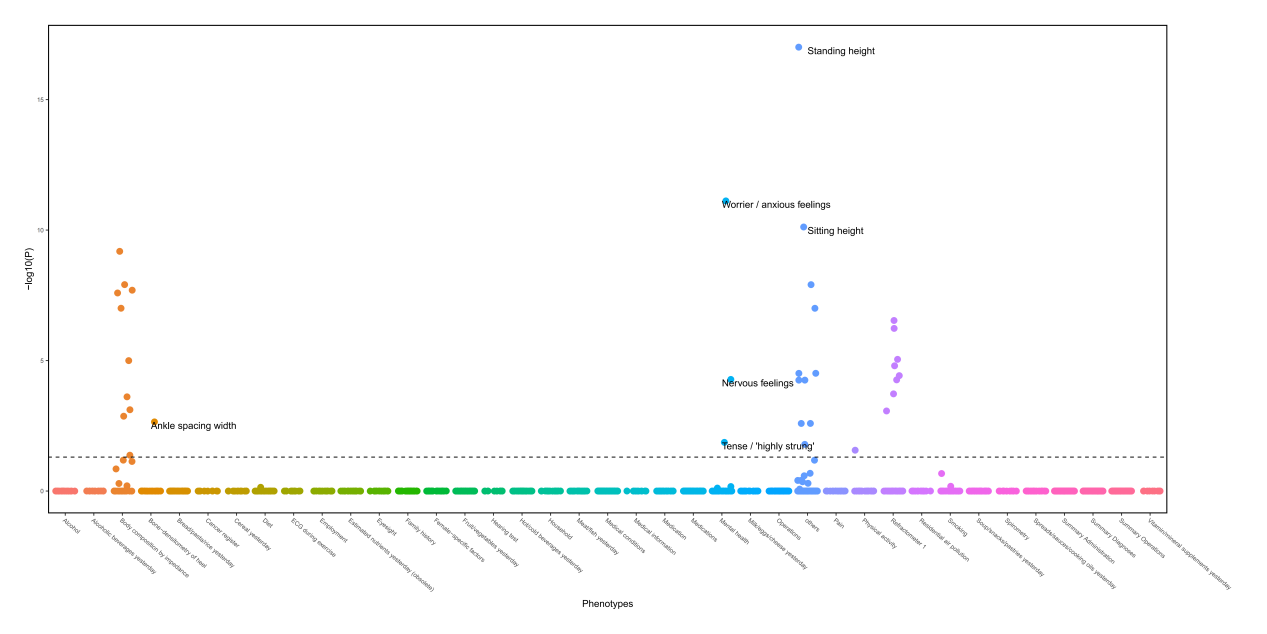


**Figure S4**

Phe-MR results, ITIH4 (whole blood) as exposure, 2514 phenotypes from UKBB GWAS as outcome, dotted lines represent Bonferrion correction thresholds, specific positive results can be seen in Table S13.
